# Supplementary material for: Evaluating privacy leakages in LLM-driven ambient clinical documentation
Source: Front Digit Health. 2026 Mar 31;8:1761624. doi: 10.3389/fdgth.2026.1761624 (PMC13076117; doi:10.3389/fdgth.2026.1761624)
Supplement: Supplementary file 1 [file Datasheet1.pdf]

Supplementary Material

1 DETECTOR VALIDATION

1.1 Sensitivity Analysis

Model	τ=0.30	0.35	0.40	0.45	0.50
Claude-3.5-Haiku	4.9	3.0	2.0	1.2	0.6
Claude-3.5-Sonnet	7.8	5.4	2.9	2.0	1.3
LLaMA-3.1-70B	17.4	13.1	9.3	6.0	3.8
Mixtral-8x7B	28.2	21.6	15.7	10.2	6.4
LLaMA-3.1-8B	30.7	23.1	17.0	10.4	6.1
Mixtral-8x22B	30.9	25.5	18.7	11.5	7.8

Table S1. Specific leakage rate (%) by model and detection threshold τ. Model ranking is stable across all thresholds.

1.2 Error Analysis

To characterize detector precision, we drew a stratified random sample of 100 positive detections balanced across the information topics, experimental conditions, and model types. Each sample was annotated by a researcher with clinical natural language processing experience, classifying detections as true positive (genuine irrelevant third-party personal information leaked from the transcript into the note) or false positive. To estimate recall, we separately drew a stratified random sample of 100 instances from the negative detection pool (cases where the detector found no leakage) and had the same annotator review each for missed leaks. Of 100 negatives, 16 were judged to be false negatives (missed leaks), yielding an estimated false negative rate of 16% (95% CI [10%, 24%]).

Information Topic	n	TP	Precision
Mental health	16	16	100%
Political views	14	14	100%
Religious views	14	14	100%
Sexuality	14	13	93%
Lifestyle	14	11	79%
Relationships	14	10	71%
Health status	14	8	57%
**Overall**	**100**	**86**	**86%**

Table S2. Detector precision by information topic ($n = 100$ sampled positive detections).

2 STATISTICAL ANALYSIS

2.1 Between-model Leakage Rates

Table S6 reports specific leakage rates by model, averaged across all experimental conditions (privacy instruction × note structure), with 95% confidence intervals computed using the t-distribution with 9 degrees of freedom.

Condition	n	TP	Precision
Priv, No SOAP	21	21	100%
No Priv, No SOAP	35	32	91%
No Priv, SOAP	29	23	79%
Priv, SOAP	15	10	67%

Table S3. Detector precision by experimental condition.

Information Topic	n	FN	FN Rate
Health status	14	7	50%
Religious views	14	2	14%
Sexuality	14	2	14%
Mental health	14	2	14%
Relationships	15	2	13%
Lifestyle	15	1	7%
Political views	14	0	0%
**Overall**	**100**	**16**	**16%**

Table S4. False negative rate by information topic ($n = 100$ sampled negative detections).

Condition	n	FN	FN Rate
No Priv, SOAP	24	6	25%
No Priv, No SOAP	27	6	22%
Priv, No SOAP	24	3	13%
Priv, SOAP	25	1	4%

Table S5. False negative rate by experimental condition.

Model	Specific leakage rate	95% CI
Claude-3.5-Haiku	2.0%	[0.4%, 3.7%]
Claude-3.5-Sonnet	2.9%	[1.6%, 4.3%]
LLaMA-3.1-70B	9.3%	[5.7%, 12.8%]
Mixtral-8x7B	15.7%	[8.5%, 22.8%]
LLaMA-3.1-8B	17.0%	[13.6%, 20.3%]
Mixtral-8x22B	18.7%	[11.0%, 26.3%]

Table S6. Specific leakage rate (%) by model, averaged across conditions. Confidence intervals are computed from encounter-level means ($n = 10$).

2.2 Pairwise Model Comparisons

Table S7 reports pairwise comparisons of specific leakage rates between all model pairs, using exact permutation tests (two-sided; 1,024 permutations) on encounter-level paired differences ($n = 10$). p-values are corrected for 15 comparisons using the Holm-Bonferroni method.

2.3 Treatment Effects

Table S8 reports the results of exact permutation tests (two-sided; 1,024 permutations) for the effects of privacy instructions and note structure on specific leakage rates. All inference is conducted at the encounter level ($n = 10$); within-encounter observations are averaged to produce one paired difference per encounter. Confidence intervals are computed using the t-distribution with 9 degrees of freedom.

Model 1	Model 2	Diff (pp)	p_{adj}	Sig.
Claude-3.5-Haiku	Claude-3.5-Sonnet	0.9	.375	
Claude-3.5-Haiku	LLaMA-3.1-70B	7.3	.029	*
Claude-3.5-Haiku	Mixtral-8x7B	13.6	.035	*
Claude-3.5-Haiku	LLaMA-3.1-8B	14.9	.029	*
Claude-3.5-Haiku	Mixtral-8x22B	16.7	.029	*
Claude-3.5-Sonnet	LLaMA-3.1-70B	6.4	.035	*
Claude-3.5-Sonnet	Mixtral-8x7B	12.7	.041	*
Claude-3.5-Sonnet	LLaMA-3.1-8B	14.0	.029	*
Claude-3.5-Sonnet	Mixtral-8x22B	15.8	.029	*
LLaMA-3.1-70B	Mixtral-8x7B	6.4	.146	
LLaMA-3.1-70B	LLaMA-3.1-8B	7.7	.029	*
LLaMA-3.1-70B	Mixtral-8x22B	9.4	.047	*
Mixtral-8x7B	LLaMA-3.1-8B	1.3	1.000	
Mixtral-8x7B	Mixtral-8x22B	3.0	.375	
LLaMA-3.1-8B	Mixtral-8x22B	1.7	1.000	

Table S7. Pairwise comparisons of specific leakage rates via exact permutation tests on encounter-level paired differences ($n = 10$). p_{adj}: Holm-Bonferroni corrected.

Effect	Mean diff (pp)	95% CI (pp)	Direction	p
Privacy instruction	-7.2	$[-9.6, -4.8]$	10/10	.002
Note structure instruction	-4.2	$[-7.0, -1.4]$	9/10	.012

Table S8. Treatment effects on specific leakage rate, estimated via exact permutation tests on encounter-level paired differences ($n = 10$). Direction indicates the number of encounters showing the expected effect direction (reduction for privacy and note structure).

3 TARGETED PROMPTING EXPERIMENT

To test whether more directly targeting third-party content improves the two-step edit approach, we tested two additional prompt variants across all six models: P3 (Targeted): “Please edit this note to preserve third-party privacy”; and P4 (Explicit): “Please remove all references to people other than the patient from this note.”

	Any Leak				Specific Leak			
Model	P1	P2	P3	P4	P1	P2	P3	P4
LLaMA-3.1-8B	.050	.326	.310	.336	.010	.179	.162	.160
LLaMA-3.1-70B	.057	.195	.226	.126	.000	.071	.081	.012
Mixtral-8x7B	.021	.186	.190	.164	.014	.076	.107	.081
Mixtral-8x22B	.033	.221	.243	.093	.005	.124	.129	.012
Claude Haiku	.029	.119	.098	.095	.000	.031	.036	.005
Claude Sonnet	.033	.176	.160	.143	.010	.048	.048	.029
**Mean**	**.037**	**.204**	**.204**	**.160**	**.006**	**.088**	**.094**	**.050**

Table S9. Any-leak and specific-leak rates by model and two-step edit prompt variant ($n = 420$ notes per cell). P1 = Specific (“only include information relevant to the patient’s care”), P2 = Generic (“preserve privacy”), P3 = Targeted (“preserve third-party privacy”), P4 = Explicit (“remove all references to people other than the patient”).

Metric	Comparison	Mean diff	Direction	p
Any leak	P3 vs P2	+0.000	3:3	.969
Any leak	P4 vs P2	−0.044	5:1	.063
Any leak	P1 vs P2	−0.167	6:0	.031
Specific	P3 vs P2	+0.006	1:4*	.500
Specific	P4 vs P2	−0.038	5:1	.063
Specific	P4 vs P3	−0.044	6:0	.031
Specific	P1 vs P2	−0.082	6:0	.031

Table S10. Pairwise comparisons between prompt variants (exact permutation test on model-level paired means, $n = 6$). *One model showed an exact tie (difference = 0); direction counts exclude ties.

4 FAILURE MODE ANALYSIS

We analyzed content changes to characterize potential failure modes of the main generate-then-edit approaches. The rule-based post-edit approach can only exhibit over-removal as it can only apply delete operations; by contrast, the two-step prompting approaches (P1, P2) can introduce new information.

4.1 Over-removal

For the rule-based "Other" section removal, no over-removal was observed on any model. For both rule-based and prompt-based approaches, the note quality metrics in Tables 7 and 8 (respectively), in particular Completeness, confirm that editing did not systematically degrade clinically relevant content.

4.2 Spillage

We define spillage as new irrelevant third-party personal information introduced by the edit step. Across 5,040 edited notes, 115 (2.3%) triggered a new detection absent from the base note (P1: 0.5%, P2: 4.1%). P2's higher rate is consistent with its tendency to rephrase rather than remove content, which can shift semantic similarity to different detector queries.

We note that in our work, this phenomenon is architecturally constrained: as the two-step pipeline feeds only the base (unedited) note into the edit step, the editing model has no access to third-party information beyond what is already present. As such, these instances reflect changes in how the detector responds to rephrased content rather than genuinely new private information. However, in setups where the editing model has access to additional context (e.g., the original full transcript, patient records), spillage represents a genuine privacy risk, as the model could reintroduce information that was absent from the base note.

4.3 Hallucination

To assess whether the editing step introduced factually inconsistent clinical content, we drew a stratified random sample of 100 edited notes (balanced across models and prompts) and compared each against its base note. Of the 100 samples, three edited notes contained information absent from the base note.

In particular, all three involved the model *substituting* a specific clinical fact with a different but plausible-sounding alternative rather than simply removing it: in one case, a malignant finding (DCIS) was mischaracterized as benign during generalization; in the other two, specific treatment plan items were replaced with more conventional-sounding recommendations (e.g., "whole blood transfusion" replaced with "physical therapy and exercises").

These errors are fluent and contextually coherent, making them difficult to detect and posing a direct patient safety risk if the edited note is accepted without thorough clinician review. We expect that providing

explicit guidance (such as on what to rewrite and how to balance privacy and clinical utility) would reduce such errors, but their occurrence underscores the risks of prompting-based approaches deployed without carefully engineered safeguards, structured evaluation, and clinician oversight.

5 EXPLORATORY ANALYSIS

5.1 Potential Effects of Documentation Requirement on Leakage

System prompt
You classify paragraphs from clinical notes into SOAP-like roles.

For each paragraph, choose ONE label that best describes its FUNCTION:
- subjective: patient-reported symptoms, history, story, feelings, psychosocial context, social history, family context, partner/child mental health, lifestyle, work stress, etc.
- objective: clinician observations, physical exam findings, vitals, test or imaging results.
- assessment: clinician's diagnostic thinking, impressions, differential diagnoses, summary of what's going on (e.g., ”likely osteoarthritis”, ”consistent with...”).
- plan: treatments, medications, investigations, referrals, follow-up, patient instructions.
- mixed: paragraph clearly contains a substantial mixture of two or more of the above roles.
- header: a section heading or subheading, such as ”Subjective:”, ”Objective”, ”Past Medical History”, ”Social History”, ”PLAN”, etc., which is very short and acts as a title rather than full content. Use ”header” ONLY if the ENTIRE paragraph is a short title-like line without full sentences.
- other: content that does not fit SOAP roles well (e.g., billing codes, metadata, templates) and is not just a header.
- uncertain: if you truly cannot decide.

Important:
- Many notes are NOT explicitly formatted in SOAP. In that case, classify the paragraph by the SOAP role it MOST CLOSELY resembles.
- If a paragraph contains a header line (like ”OBJECTIVE:”) AND additional clinical sentences
or exam details, do NOT label it ”header”. Instead, label it according to the main content (e.g., ”objective”, ”assessment”, ”plan”, or ”mixed”).
- Always return ONLY a JSON object with a single key ”label”.

User prompt
Classify the SOAP-like role of this paragraph:

```
<<<PARAGRAPH_START
{paragraph}
PARAGRAPH_END>>>
```

Figure S1. Prompts used for SOAP-like paragraph classification.

We conducted a small post-hoc analysis to explore why the tested closed models (Claude-3.5-Haiku, Claude-3.5-Sonnet) were found to leak more information when instructed to generate notes in the SOAP structure.

Specifically, we explored where the detected leakage sentences were positioned within each generated note. We first split each note into paragraphs using newline-based heuristics, then prompted GPT-4o-mini to classify whether the segment belonged to a Subjective-like section (see Figure S1), and finally checked

whether a note sentence with leaked information originated from a paragraph classified as similar to content in a typical Subjective section.

Using this method, for the six models (2 closed, 4 open), we computed for each model the proportion of detected leakage sentences assigned to Subjective-like sections under SOAP (p_{SOAP}) and non-SOAP (p_{noSOAP}) conditions and the difference $\Delta = p_{\text{SOAP}} - p_{\text{noSOAP}}$.

The observed difference in mean p_{SOAP} between the two closed Claude models and the four open models was $D_{\text{obs}} = 0.148$, and the difference in mean Δ was $\Delta_{\text{obs}} = 0.508$.

To contextualize these values, we performed a two-sided model-level permutation test using exact enumeration: the two closed labels were reassigned across all $\binom{6}{2} = 15$ possible partitions of the six models (leakage data held fixed), and D and Δ were recomputed for each. The resulting exact permutation p-values were 0.467 (7/15) for D_{obs} and 0.067 (1/15) for Δ_{obs}. We note that with only six models in a 2-versus-4 split, the minimum achievable two-sided p-value is $1/15 = 0.067$; the observed interaction is the most extreme value in the permutation distribution but cannot reach conventional significance under this design.

In short, we found that only the Claude models showed an increase in the proportion of leakages assigned to Subjective-like sections under SOAP prompts relative to non-SOAP. While this analysis is not inferential given the small number of models and examples, it lends support to our conjecture that helpfulness-aligned models keenly aware of section requirements can overgeneralize to the point of including irrelevant personal information. We leave exploring the exact mechanism between note requirement understanding and overgeneralization to future work.
